# Supplementary material for: Investigating Bacillus anthracis genomic diversity and trait-specific lineages in an endemic area in northern Tanzania through a combination of traditional and culture-free sequencing approaches
Source: PLoS Negl Trop Dis. 2026 Aug 10;20(8):e0014611. doi: 10.1371/journal.pntd.0014611 (PMC13489486; doi:10.1371/journal.pntd.0014611)
Supplement: Supplementary file 1 [file pntd.0014611.s001.docx]

# **S1 Appendix. Supporting Information for “****Investigating Bacillus anthracis genomic diversity and trait-specific lineages in an endemic area in northern Tanzania through a combination of traditional and culture-free sequencing approaches”**

## Text, Tables and Figures

#### **Text A. Classification of sequences in clusters 3.1 and 3.2**

One targeted capture (TC)-derived sequence from a sheep in the NCA, denoted AN16-145-S_S2 collected in 2016, was placed within the NCA-clade, but showed unusual SNP markers that deviated from the system proposed by Bruce et al. (2020). Although the sequence was identified as part of the 3.2 cluster, it lacked the expected allele at the SNP position that defines the 3.1 cluster. By definition, the 3.2 cluster is nested within the 3.1 cluster, carrying the 3.1 defining SNP (CP 3086718) and an additional site characterising it as 3.2 (CP 3366432). Consequently, the SNP pattern encountered in the sequence in question is counterintuitive, showing the marker on the lower, but not the upper hierarchical typing level. When the read data for both cluster-defining SNP sites was inspected, each site had read counts for exactly two alleles. The differences in read counts for the formally called allele and the alternative allele were <50 reads (Table S1). We hypothesise that this animal harboured isolates of both types in similar proportions, making it difficult to determine the consensus genotype using the TC approach.

Table A: Overview of the expected and observed base alleles for classification of *Bacillus anthracis* genomes into clusters previously defined by Bruce et al. (2020). NA – not applicable, CP – chromosomal position

| SNP site of interest | Combination 3.1 cluster | Combination 3.2 cluster | Observed combination of allele calls | Observed read counts,  allele (count) |
| --- | --- | --- | --- | --- |
| SNP site 1  (CP 3086718) | C | C | A [would define out of 3.1] | A (135), C (97) |
| SNP site 2  (CP 3366432) | NA | C | C [would define within 3.2] | C (75), T (33) [ ‘T’ would define it as out of 3.2] |

### **Supplementary figures**

Fig A: Schematic representation of steps taken to test for statistical difference in mean SNP distances between sequences derived from isolate culture and from culture free targeted capture. Subsetting sequence data was used to match the number of cases between both sequence retrieval method and repetition of subsetting was used to account for heterogeneity in number of sequences per case as well as sequence diversity. The distribution of difference between the means was assessed across 10,000 permutations (see Fig E).

**Fig B: Schematic representation of the zone of high anthrax infection risk within the Ngorongoro Conservation Area**. For the original map and study which incorporates much greater detail, please see Aminu et al. (2022)

**Fig C: Minimum spanning tree of Bacillus anthracis genomes from the Ngorongoro Conservation Area comprising cluster 3.2 (n = 206).** Tree is the same as that shown in Figures 1 and 6 in the main manuscript. Size of the node corresponds to the number of identical genomes, as indicated by the legend. Branch lengths (in single nucleotide polymorphisms – SNPs) are indicated by the example arrow shown. Genotypes are as described in Forde et al., 2022. This figure was generated using the web-based tool MSTree V2, available at https://achtman-lab.github.io/GrapeTree/MSTree_holder.html.

***Fig D: Maximum likelihood phylogeny of* Bacillus anthracis *genomes from the Ngorongoro Conservation Area and broader Arusha and Kilimanjaro regions analysed in this study (n = 213) combining data from Figures 1 and 6, along with full genotype and sequence annotations.*** *Isolate-derived sequences are shown by circles, whereas targeted-capture-derived sequences are shown by squares at branch tips.*

**Fig E:** **Visualisation of permutation tests for the difference in single nucleotide polymorphism (SNP) distances between targeted capture (TC) and isolate-derived sequences, applied to 100 data subsets sampled from sequences in the NCA-clade**. Histograms show the distribution of differences between means of SNP distances between TC and isolate-derived sequences after permutation. 100 datasets from the TC-derived sequences were subsampled to represent 55 cases per dataset. These were compared against the SNP-distances between sequences derived from 55 cases by isolate culture. The observed difference in means is indicated as a red line.

**Fig F: Ridgeplots visualising the number of ambiguous sites and gaps among Bacillus anthracis sequences from different sequence retrieval methods**. While there is a tendency for more gaps and more strongly so for ambiguous sites among sequences derived from targeted capture (TC), the majority of sequences from either method show a similar frequency of both site-types.

**Fig G: Mean number of single nucleotide polymorphism (SNP) differences between Bacillus anthracis sequences of case pairs from the Ngorongoro Conservation Area (NCA) and surrounding regions (n_total_ = 213) between cases**. A) Comparisons between sequences belonging to Bruce cluster 3.1, collected from carcasses and cases outside the NCA in the Arusha (n = 5) and Kilimanjaro regions (n = 2). B) Comparisons between sequences belonging to Bruce cluster 3.2, collected from carcasses and cases within the NCA (n= 206).

**Fig H: Mean number of single nucleotide polymorphism (SNP) differences between Bacillus anthracis sequences from the Ngorongoro Conservation Area (NCA) and surrounding regions (n_seq.total_ = 213), within and between cases**. A) Mean SNP distances within cases with more than one sequence; B) Mean SNP distances from comparisons between the sequences of all case pairs in the full dataset; C) Mean SNP distances from comparisons between sequences of case pairs belonging to Bruce cluster 3.1, collected from outside the NCA in the Arusha and Kilimanjaro regions; D) Mean SNP distances from comparisons between sequences from case pairs belonging to Bruce cluster 3.2, collected within the NCA

**Fig I:** **Visualisation of permutations test for the differences between single nucleotide polymorphism (SNP) distances among sequences sampled as part of an epidemiologically linked group (ELG) and those not linked to any other cases**. Histograms show the distribution in difference of means among the permutations; the observed difference in means is indicated as a red line. A) Using the full dataset. B) Using sequences of the Ngorongoro Conservation Area clade only, excluding those sequences sampled in the neighbouring regions.

**Fig J: Visualisation of permutations test for the differences between single nucleotide polymorphism (SNP) distances among sequences sampled within and outside the high risk zone in the Ngorongoro Conservation Area and neighbouring regions.** Histograms show the distribution in difference of means among the permutations; the observed difference in means is indicated as a red line. A) Sequences assigned ‘outside of the high risk zone’ were used including those sequences sampled outside of the NCA. B) Sequences assigned ‘outside of the high risk zone’ were used excluding those sequences sampled in the neighbouring regions.
