# Supplementary material for: Investigating Bacillus anthracis genomic diversity and trait-specific lineages in an endemic area in northern Tanzania through a combination of traditional and culture-free sequencing approaches
Source: PLoS Negl Trop Dis. 2026 Aug 10;20(8):e0014611. doi: 10.1371/journal.pntd.0014611 (PMC13489486; doi:10.1371/journal.pntd.0014611)
Supplement: Supplementary file 2 [file pntd.0014611.s005.docx]

We thank the editor and reviewers for their constructive suggestions for improving our manuscript. We provide detailed responses to each of the comments below (bold text).

We have further added the numbers of the comments directly into the clean main text document.

Reviewer 1

1. The manuscript is very extensive. The authors conducted a detailed analysis of their data. The text does mostly a good job in explaining what has been done and found. However, in my opinion the authors have failed in presenting a capturing story that readers with lower capabilities of deep (bioinformatic) analysis can (or are willing to) follow. This is mostly owed to the authors’ decision to use the (non-canonical) B. anthracis typing nomenclature of Bruce et al. (2020) (reference 23). While this is (of course) not wrong or illegitimate, it is not helpful for easy understanding of the study’s SNP-diversity-topic. The most widely accepted, and in my opinion most useful, canonical SNP-typing system for the analysis at hand, is that of Sahl et al. 2016 (reference 13). Only using this system, coupled with ideal visual (bioinformatic) aids such as minimum spanning trees (MST) based on SNP analysis will provide a straightforward understanding of the central points of the authors’ work. The authors are referred to some examples on how to compile a clear graphical output here: https://pmc.ncbi.nlm.nih.gov/articles/PMC8218748/ (Figs. 2-4)), https://pmc.ncbi.nlm.nih.gov/articles/PMC10610006/ (Figs. 2-4), or https://pmc.ncbi.nlm.nih.gov/articles/PMC12566857/ (Figs. 3-6). In such MST figures the SNP-diversity becomes immediately obvious.

**Thank you for your suggestions in making our narrative clearer. However, the suggested typing scheme by Sahl et al. as proposed in their 2016 work, would not provide the differentiation between strains that we aim to demonstrate in our current study. As we have outlined, all our sequences fall within the Ancient A subclade; we have added a contextualising tree as a panel to Figure 1 ( (line 446) to make the taxonomic placement of our samples clearer to the reader (see response to comment 2 below). When comparing phylogenies constructed by van Ert et al. in 2007, the supplementary figures 2 and 3 of Sahl et al.’s 2016 work and then Bruce et al.’s system as published in 2020, we can see clearly how typing schemes have gradually become more detailed. This applies especially to Bruce et al.’s differentiation of what they call primary cluster 3, which broadly corresponds to the Ancient A-clade. It was our aim to go beyond this level and to assess all detectable diversity at even finer resolution and at a local scale. Applying Sahl et al.’s (2016) typing would not add substantial benefit over the Bruce typing scheme we used, as it cannot distinguish genetic diversity at such fine scales. Similarly, in regards to the minimum spanning tree, we argue that the presented phylogeny, based on the whole genome SNP alignment, is an appropriate and well-established way to visualise the distances between sequences. The minimum spanning trees presented in the references listed by reviewer 1 all describe samples collected at much greater geographic distance, spanning different countries and large distances within countries. This greatly exceeds the dimensions of the Ngorongoro Conservation Area, and such geographic distance is likely to also be reflected in greater genetic distances (as also demonstrated in our analysis by the separation of the NCA-clade from sequences of the Arusha regions) and thus more resolved trees. Based on this we see limited benefit of showing a minimum spanning tree as one of our main results in addition to our wg-SNP phylogeny in the main manuscript. However, we have added a minimum spanning tree to the supplementary material (Figure S3) to contextualize our findings with the representations exemplified in the cited literature. To ensure that results can be linked across different typing schemes, we have also clarified that the Ancient A clade was referred to as canSNP group A.Br.034 in Sahl et al (line 102).**

2. More, there is no phylogenetic tree showing the placement of the isolates within the global diversity of B. anthracis. This tree can be centered (largely reduced) to the A.Br.034 clade into which the new isolates fall. Also, I suggest collapsing “mini”-lineages of isolates showing less than 5 SNP differences among their closest relatives. This tree can be easily combined into a single figure with the one described in the previous comment

**An updated Figure 1 (line 446) has been created to provide more global context for our phylogeny, as suggested. Please also see response to comment 3 below.**

3. This leads me to the second major issue of this manuscript. It is well understood in the B. anthracis research field that differences of only a few (<4, max. 5 SNPs) between isolates are regarded as of low significance (also compare https://pmc.ncbi.nlm.nih.gov/articles/PMC8218748/ ). Therefore, I would be cautious to call widespread differences of 1-2 SNPs among strains “high genetic diversity”.

**Addressed in combination with comment number 10 by reviewer 1 and comment number 40 by reviewer 3.**

Comment number 10 “437-438: I find it remarkable that “Forty-four cases were represented by more than one sequence (range: 2-6, median 3)”. But then these sequences only differ in very few SNPs. Again, why should we bother and unnecessarily bloat the SNP-diversity?”

Comment number 40 “The authors report 104 SNP profiles that differed by at least one SNP. Is a difference of one SNP sufficient as a cutoff to distinguish strains? In other bacterial studies, thresholds of 3–5 SNPs are often used. Please justify the selected cutoff or provide supporting references.”

**Thank you for your interest and concern. The 5 SNP cut-off used in Abdel Glil 2021 was intended to distinguish between outbreaks. We deliberately avoided defining outbreaks *a priori*, and instead asked what levels of genetic divergence are observed when grouping sequences by their geographic and temporal proximity, making them a potentially "epidemiologically linked” group, ELG. In fact upon inspecting our data in the context of these queries, we found 20 out of 22 ELGs contained sequence pairs with >5 SNP distances between them.**

**Our study’s aim, however, was to comprehensively describe genetic diversity across physical scales, and to assess differences between sequences derived from culture and culture-free target enrichment. Therefore, complete reporting of all SNP differences without prior categorization was crucial. Consistent with our previous work (Forde et al.) we found identical SNP profiles across cases that were separated by months and in some cases years. And conversely, in other instances sequences detected within the same case differed by up to 32 SNPs. The fact that seemingly unrelated cases can be caused by anthrax strains that are genetically indistinguishable, strongly suggests that even minimal differences involving a single SNP can be biologically meaningful when it comes to reconstructing potential transmission links which gave rise to our understanding of the findings as “high diversity”. Moreover, many of these new minimally divergent SNP profiles were recovered more than once and by multiple methods (culture/target enrichment), adding support to these being genuine variants. We carefully discuss the evidence for quantifying genetic variation down to single SNP differences as well as the potential limitations of our approach (sections at lines 756 and also 819 in the discussion).**

4. Though I congratulate the authors for retrieving SNP-typing compatible data from culture-free target-capture, I have considerable reservations for the wide-spread use of this approach for B. anthracis. This is because (and the authors even correctly address this challenge in their Discussion section) there are usually large numbers of closely related Bacillus cereus sensu lato organisms in soil alongside B. anthracis. Often these bacteria feature genomes that are extremely similar to that of B. anthracis. Only long-read genome sequencing techniques can (all but) avoid trans-species DNA assemblies, possibly leading to fictitious SNPs. Read lengths generated in the study at hand were 2x75 or 2x100 bp only, which complicates correct attribution of reads. Indeed, 75 auf of 288 sequences were excluded from further analysis because of quality issues.

**We have adjusted the discussion to consider these useful points and have tried to clarify potential limitations of the TC method (lines 797-807).**

**We would like to clarify that no soil samples were processed using TC in this study (also see response to point 5 below). While we cannot rule out that samples we included in TC sequencing, namely blood, tissue and swabs, may have had some contamination from other sources, the presence of soil-derived *Bacillus* species is unlikely to have been a major factor in our data.**

5. Finally, because of these challenges it would have been prudent to perform a proof-of-principle experiment beforehand in order to check if different genotypes can be identified by a TC sequencing approach in soil at all and at which quality-level. For this, B. anthracis negative soil taken from Ngorongoro would be spiked with spores of two different B. anthracis strains (e.g., Sterne and another vaccine strain such as Pasteur) and genotyped using TC followed by DNA-sequencing.

**We support that in theory a proof of principle experiment would have added a valuable angle to our findings. However, there may be a potential misunderstanding affecting this suggestion, as indicated through Reviewer 1’s summary of our work. The summary states “Herein, the authors employed classical culturing and array-based DNA enrichment from soil samples of a prominent (hyper)endemic area.” This suggests that we worked only or mainly with soil samples, which is not the case as can be viewed in the metadata stored in the Microreact project: 19 out of 213 samples were collected from soil and none of these were processed with TC, but instead all 19 soil derived genomes were generated using isolate culture.**

6. Line 102: reference 22 should be 13 (A.Br.AncientA was unknown in 2007).

**Addressed as suggested**

7. L 198: <https://pmc.ncbi.nlm.nih.gov/articles/PMC8218748/>could be added as a reference

**We are not certain that the stated line is the one truly meant but assume that instead L108 (now line 95) was meant. We have added this reference as suggested.**

8. L 311, better: “…profile of any strain differs…”.

**Addressed as suggested. (line 308)**

9. 363-369: I find it problematic to separate anthrax outbreaks by the parameter “time”: spores remain viable in soil for years possible decades and, are thus occasionally the source of recurring outbreaks of the same genotype (give or take a handful of SNPs).

**(Now at 367-371) We agree with the reviewer that this is the case. However, between sporadic cases, anthrax cases often appear as “outbreaks” of spatio-temporally clustered cases. We examined whether such clustered incidences were associated with the same or multiple different BA strains. Our evidence points to the latter.**

10. 437-438: I find it remarkable that “Forty-four cases were represented by more than one sequence (range: 2-6, median 3)”. But then these sequences only differ in very few SNPs. Again, why should we bother and unnecessarily bloat the SNP-diversity?

**(Lines referred to in the comment now at 425-426) We would like to clarify that ‘more than one sequence’, as cited from our manuscript, only refers to the number of sequences generated, not to the number of different SNP profiles observed among these sequences. Please see response to comment number 3 above for our justification why even small numbers of SNP differences, down to single SNP, are potentially informative. Our previous work (Forde et al. 2022) further demonstrated that differences >1 SNP are highly unlikely to have arisen over the course of a single infection, which implies that such differences within the same host are likely caused by a diverse inoculum. This highlights the importance of documenting genetic diversity at such fine resolution.**

11. L 500-503: this mini-paragraph is hard to understand. Please revisit.

**Addressed as suggested. (line 495-498)**

12. L511-512: did the authors consider (slightly) higher SNP differences identified in their culture-derived sequences because these organisms were propagated in in-vitro cultures for several extra generations (DNA replications) while the TC-derived sequences were not?

**We have added a brief discussion of this finding in the discussion at lines 767-775.**

13. Legend of Fig. 1 is odd: “All NCA sequences fell within cluster 3.2 defined by Bruce … corresponding to the 3.1 cluster.” This is Results and thus should not appear in a figure legend.

**Addressed as suggested. (lines 448-460)**

14. Similar in legend of Fig.2: “While the curve for isolate-derived sequences …sufficiently capture the SNP profile diversity.” This is Results and thus should not appear in a figure legend.

**Addressed as suggested. (line 518-521)**

15. Also, header of table 2: “In three of four cases…both methods.” This is Results and thus should not appear in a table header.

**Addressed as suggested. (lines 529-530)**

16. Finally, in legend of Fig.3: “Fewer SNP differences separate the sequences … over 250 SNPs.” This is Results and thus should not appear in a figure legend.

**Addressed as suggested. (lines 579-583)**

17. L575 vs last row of Table 4: “28 vs “32” is this correct? Should it not be the same numbers? Did I miss something?

**We believe the confusion might stem from the subtle difference between within-case and within-sample assessment. The table (line 569) shows the values for within-case distances (44 cases), while the text at line 564-567s reports the assessment of within-sample distances (13 cases). We chose to report the within-sample distances separately, as a lot fewer datapoints were used than for all subsets presented in the table.**

18. L 802 and 816: https://pubmed.ncbi.nlm.nih.gov/23937393/ should be added as a new reference.

**Thank you for this relevant reference, we have added it as suggested (now Ref 73) at line 826, as well as in 839.**

Reviewer 2

19. I am curious as to the justification for excluding PXO1 and PXO2 from the target capture. One could make the argument that PXO1 and PXO2 are also 'core' genomic regions in BA though not chromosomal. While it would be a small addition to the total genome sequence length nevertheless genomic diversity between lineages in the PXO1/2 regions would be interesting to investigate. Alternatively, baits capturing non-anthracis bacillus species could also lead to false-positive novel SNP profiles, which can be mitigated by confirming the presence of PXO1/2 regions (Keeping in mind that B. Tropicus for eg may also carry the anthrax plasmid https://www.biorxiv.org/content/10.1101/2023.12.20.572685v1 ).

**Answered in combination with comment number 20 by reviewer 2, see below.**

20. In addition, some discussion on where the SNP differences between the SNP profiles located on the genome would be useful. Are they randomly distributed or are there hotspots? What is the nature of the mutations (eg: are they mostly non-synonymous?)

**We agree with the reviewer that exploring the diversity within the PXO1/2 regions would be interesting, as would describing the distribution of SNPs across the genome. These topics will be addressed in a separate publication (in prep) based on the culture-derived isolates of this dataset. When designing the panel for targeted capture (Dennis et al., 2022), we chose to omit the plasmid sequences, as we were less certain of their suitability for phylogenetic inference, and we were limited by the cost incurred with increasing the number of ‘baits’.**

21. L486 - 491: If this rarefaction analysis was performed across all publicly available anthrax genomes, what would be the rate of discovery of new SNP profiles per new genome added? How does that compare to the rate observed in this study for isolate derived and TC-derived? This would contextualize the genomic diversity of BA in the NCA from a global perspective

**(Lines 325 – 328) To our knowledge, a global study that comprises all available whole genomes of *Bacillus anthracis* is not readily available for comparison, and it is not possible to predict what that curve would look like. However, given the geographical distinction between the 3.2 and 3.1 clusters (NCA vs. non-NCA clade) in our study, we have reason to believe that attempting to comprehensively sample genetic diversity is (if at all) more achievable in an endemic, localized area. While we cannot predict the outcome of a rarefaction analysis on a global BA dataset, we must assume that sampling coverage for any given area would be much lower and that much larger sample sizes would be required before the rate of discovering new types would decline i.e. for the curve to level of.**

22. L87: "blood stream" --> "bloodstream"

**Addressed as suggested. (line 87)**

23. L259: 111 TC (L233) + 51 (L223) isolates in this study + 72 + 52 from another study totals to 286, not 288. Please double check the numbers and restate them in this section to make it clearer.

**Thank you very much for spotting this error (corrected value at line 258) and our apologies for the oversight. The “missing” two sequences were part of the data processed for the Dennis et al. 2022 publication. They were removed from out dataset due to not meeting the quality requirements for sequences in this study and this is where their categorization got mixed up during writing (‘included in initial dataset, removed during quality testing’, instead of ‘not included in the first place’).**

24. L286 - L287: 'Error! Reference source not found' in text

**(Now at line 283) Thank you for pointing this out. This error did not appear when viewing the documents in our desktop or web-based Word applications. To be safe, we have exchanged the link for a new one. We would like to ask the editors whether this issue might have occurred through any reformatting, potentially relating to PDF exports? We have encountered this issue at other submissions with other journals before and while we hope that renewing the link will have fixed the problem, this issue might occur after upload and might not be in our full control to avoid.**

25. Outline how only polymorphic sites were extracted from the alignment. Specify the values supplied to the IQ-TREE -fconst parameter

**For the first part of your question, we would kindly point to lines 265-277 in the methods, where we have tried to detail how this implemented. If there are further questions about this SNP calling process, we would kindly ask for further guidance on which part remains unclear.**

**For the second part of the question, we applied ascertainment bias correction (+ASC) but did not include the -fconst parameter. Our primary focus was on tree topology (i.e. phylogenetic grouping, transmission patterns, and lineage structure), for which branch length estimates are of secondary importance. In this dataset, the genomes are highly conserved and the majority of splits are shallow, so omission of -fconst is expected to result primarily in a near-uniform scaling of branch lengths rather than differential distortion among clades.**

26. L328 - 336: If I have understood correctly, the authors here created random subsets of TC data and compared the mean SNP distance within each subset against them mean SNP distance between isolate derived sequences. This section can be improved for clarity (maybe a simple flow diagram can be added) as it is currently hard to follow. Was the deduplicated set of sequences used to make the different subsets?

**Yes, we confirm that the first sentence describes the process correctly as we intended. We have edited the paragraph to make the rationale and logic behind the subset generation clearer (lines 330-334). No, we intentionally used the full dataset and not the deduplicated dataset for this analysis. Multiple identical sequences from the same case are considered a plausible and expected result for either method (TC and isolate culture) and thus needed to be incorporated within the tests. We have further added a schematic outline of the subsetting process to the supplementary materials (Figure S1).**

27. L339: 'Multiple genomes' - Please state exactly how many

**Addressed as suggested. (lines 340-342)**

28. Fig 3: Might be better to show all vs. all within-case and between case SNP distances as boxplots

**Thank you for this suggestion. While we initially considered using boxplots, we argue that the key information, which is that most case comparisons show mean SNP distances comparable to the highest mean SNP distances found within cases, is conveyed well in the barplots. The additional advantage is that the information on number of sequences per case or case-pair can be displayed as the colour of the bars, while adding coloured data points to a boxplot proved visually unclear. However, we have added a new figure to the supplementary materials, now Figure S8, referred to at line 562, that shows the distribution of mean SNP distances alone, without number of sequences, as boxplot for direct comparison.**

29. Table 5: Also include between ELG SNP distances

**Addressed as suggested. (line 639)**

30. This appears to be the same tree as Fig 1, perhaps the two figures can be combined as shown in Fig S2

**(Figure at line 666) We did consider this option when preparing the original manuscript. However, we felt that the main messages shown in Figure 1 and 6 were distinct and were easier to capture when shown separately. We defer to the editor as to whether there is a preference to combine these, along the lines of Fig S4 (formerly Fig S2) (but with newly added panels in Fig 1 and omitting previously-defined genotypes), or to maintain them as separate figures.**

31. L738 - 740: 'In some cases' - please state exactly how many cases. From my understanding four matched samples were obtained out of which 3 showed agreement.

**Addressed as suggested. (line 739-742)**

32. L805: "Inherit limit" - typo, maybe the authors meant "inherent limit" ?

**Addressed as suggested. (line 829)**

33. Fig S4: The plot is not labelled correctly so there is no way to tell which is TC and which is isolate derived

**We suggest that this is likely an issue with the display tool. Opening the file in word shows the colours clearly, with TC samples in a reddish/orange colour and isolate genomes in peach analogous with colour coding in previous graphs**.

Reviewer 3

34. In the Introduction, please replace the abbreviation “BA” with B. anthracis.

**(Introduction starts at line 70) We are sorry if this presentation conflicts with the reviewer’s preferences. However, we would suggest that it is common practice that abbreviations are only written out upon first mention. We do not believe creating a mixture, in which there is a third version, *B. anthracis,* used only within the introduction would add clarity. We defer to the editor for journal-specific preferences.**

35. The Introduction is too long and should be reduced. For example, in lines 147–174, the authors provide extensive information on risk factors and ecological and environmental aspects, which is not appropriate for the Introduction section. This part should be shortened, and the detailed information should be moved to the Discussion.

Adressed in combination with comment number 37 by reviewer 3 “The authors elaborate excessively on the study objectives and attempt to justify each objective in detail. The objectives should be stated more concisely to avoid confusing readers.”

**We have shortened the respective sections in the introduction and moved some of the material to the discussion. We have further made minor edits to shorten the remainder of the introduction, and simplified the objectives in the last paragraph of the introduction. (Mainly lines 130-168)**

36. The authors elaborate excessively on the study objectives and attempt to justify each objective in detail. The objectives should be stated more concisely to avoid confusing readers.

**Addressed together with comment number 35 by reviewer 3, please see above.**

37. Materials section, line 216: “Additional DNA extracts (n = 5) from animal (4) and human (1) blood samples.” It is unclear whether (4) and (1) refer to reference numbers or to the number of samples. Do you mean (n = 4) and (n = 1), or simply four and one samples? Please clarify and revise accordingly. The same issue appears in Table 1. Please find a way to clearly differentiate reference numbers from sample numbers.

**We have addressed the first part of the comment as suggested. (line 213)**

**Regarding information in Table 1 (line 429-433): the information displayed presents inherent complexity, as we are showing cases represented by multiple genomes, stratified by method of sequence retrieval and host species. We have explained in the legend that given numbers refer to number of cases and numbers in parentheses refer to the number of genomes. Adding another layer to stratify exactly how many genomes were collected in each single case would, in our opinion, exceed the key intention of this table, which is to provide an overview. For this level of detail, we have provided the metadata in the Microreact project, where each case is listed with all its associated genomes. If the reviewer’s concern is that numbers in parentheses in Table 1 could be misunderstood as literature references, we have added a short sentence in the legend to clarify this.**

38. Figure 2 requires higher resolution

**(Figure at line 515) We have double checked the resolution of this figure, and it seems adequate from our end. The problem may therefore arise due to the format in which files have been provided to the reviewers. We are happy to work with the journal to ensure that reviewers have access to files with appropriate resolution for review.**

39. The authors report 104 SNP profiles that differed by at least one SNP. Is a difference of one SNP sufficient as a cutoff to distinguish strains? In other bacterial studies, thresholds of 3–5 SNPs are often used. Please justify the selected cutoff or provide supporting references.

**Addressed together with comments 3 and 10 by reviewer 1, please see above.**

40. A legend explaining the barplot colors is missing in Figure 4.

**(Figure at line 600) We have addressed this comment by adding text to the caption, stating that each colour represents a different epidemiologically linked group (ELG). However, we don’t believe it aids understanding of the figure or general visual readability if the numbered ELGs would be listed with their respective colours, as they are not discussed separately. The only ELG that is described in further detail is ELG-16, which is shown within its own figure (Figure 5). We have now also added demarcations (a circle in the map and a dot under the barplots) to point out ELG-16 also in Figure 4. If readers are interested to see where cases of each ELG were located, this is possible through the link to the Microreact project. We have added a sentence to the legend to clarify this option (line 604-606).**

41. Discussion, lines 718–725: “Our results show that (1) regional clustering…” It is unclear whether the numbering refers to references or to listed points, as both follow the same journal style. Please revise the format (e.g., 1), 1., [1], or a, b, c) to avoid confusion.

**Addressed as suggested. (line 719-726)**

42. Although the abbreviation TC is used throughout the manuscript, the explanation “targeted-capture (TC)-derived sequences” is provided only in the conclusion. Please define TC at its first occurrence in the manuscript.

**Please consider line 115, we believe this might have been overlooked.**
