# Supplementary material for: Investigating Bacillus anthracis genomic diversity and trait-specific lineages in an endemic area in northern Tanzania through a combination of traditional and culture-free sequencing approaches
Source: PLoS Negl Trop Dis. 2026 Aug 10;20(8):e0014611. doi: 10.1371/journal.pntd.0014611 (PMC13489486; doi:10.1371/journal.pntd.0014611)
Supplement: Supplementary file 3 [file pntd.0014611.s006.docx]

We thank the editor and reviewers for their constructive suggestions for improving our manuscript. We provide detailed responses to each of the comments below (bold text). To the editors: we have removed and adjusted the sentence containing the comment on “data not shown” (line 901).

Reviewer #1: The authors have addressed my previous remarks and concerns in their rebuttal. I still disagree with the authors’ reasoning about the negligible relevance of small SNP differences among closely related strains. However, it is fine to have different opinions, as data typically allows for more than a singular explanation. Yet, in my opinion, the most informative visual data interpretation of the work at hand (i.e., supplementary figure 3) lacks any strain designation. Please revisit and add names and SNP distances.

**We have addressed this comment by adding the genotypes annotations described by both the current and previous study (Forde et al. 2022) to Supp Figure 3.**

Reviewer #2: The authors have addressed my comments. However for comments #19, #20 (on not including PXO regions in the baits) and #25 (the justification for using +ASC instead of -fconst), while I agree with the authors justifications, I would suggest they also include it in the manuscript itself as a brief sentence (#25 in the appropriate methods section and #19 and #20 in the discussion perhaps?) as I believe they are questions other readers might also have.

**Addressed as suggested. Please see added text in lines 745-751 and 237-238.**
