# Supplementary material for: Investigating Bacillus anthracis genomic diversity and trait-specific lineages in an endemic area in northern Tanzania through a combination of traditional and culture-free sequencing approaches
Source: PLoS Negl Trop Dis. 2026 Aug 10;20(8):e0014611. doi: 10.1371/journal.pntd.0014611 (PMC13489486; doi:10.1371/journal.pntd.0014611)
Supplement: Supplementary file 4 [file pntd.0014611.s007.docx]

We thank the editor and reviewers for their constructive suggestions for improving our manuscript.

We have edited the GPS data on human cases as available through the Microreact Project ( <https://microreact.org/project/ivGWEG2R5UUM3vdQ5kpTck-ba-ncahilbig-medvecky-forde>) to a lower resolution, such that individuals are not identifiable. We have also added the information about this adjustment to the GPS section of the Microreact README file in the supplementary material.
