# Supplementary material for: Investigating Bacillus anthracis genomic diversity and trait-specific lineages in an endemic area in northern Tanzania through a combination of traditional and culture-free sequencing approaches
Source: PLoS Negl Trop Dis. 2026 Aug 10;20(8):e0014611. doi: 10.1371/journal.pntd.0014611 (PMC13489486; doi:10.1371/journal.pntd.0014611)
Supplement: Supplementary file 5 [file pntd.0014611.s003.rtf]

Supplementary Material for “Investigating Bacillus anthracis genomic diversity and trait-specific lineages in an endemic area in northern Tanzania through a combination of traditional and culture-free sequencing approaches “
Metadata displayed in Microreact Project BA_Diversity_Hilbig-Medvecky-Forde 
URL: https://microreact.org/project/ivGWEG2R5UUM3vdQ5kpTck-ba-ncahilbig-medvecky-forde
 
 
The Microreact project contains a midpoint-rooted phylogenetic tree of 214 sequences including the reference sequence of the Ames ancestor (NC_007530.2,(1)), a map of the sampling locations as well as a table of metadata. The Microreact tool provides an intuitive graphical user interface to explore phylogenetic trees with their associated metadata. Single sequences or groups thereof can be selected directly from either of these three representations or shown as the result of setting filters within the metadata table. This provides the options for exploring and viewing very specific sets of sequences under multiple filtering conditions; for example: one could choose to display only those sequences of a manually selected area that were sampled from sheep and were derived from isolate culture to then assess how many different cases these sequences belong to. Using the eye-symbol in the right-hand top corner of the window, one can adjust the display of colours on tree-tips and map-points to represent a metadata trait of their choice, with legends indicating colours associated with the respective trait levels along the right-hand side of the window. Microreact further provides several download options of the displayed data, such as Newick (phylogenetic tree), png (all visual elements) and csv (metadata table). Below we provide additional information for several of the displayed variables.
 
ELG_ID
 
Refers to the epidemiologically linked groups (ELG) that were defined as sequences associated with cases that were found within ≤ 10 km distance and ≤ 10 days between each other.
 
Method
 
Refers to the use of isolates from bacterial culture or the metagenomic targeted capture approach to prepare the sample material for sequencing.
 
Infection zone
 
Refers to the area of higher risk of infection for livestock as determined through participatory mapping within focus groups of pastoralist communities within the Ngorongoro Conservation Area (NCA), documented in a study published by Dr. O.R. Aminu (2), see supplementary material Figure S1 of this paper for a simple representation of the area. 
 
Season anthrax
 
Refers to four seasons corresponding to the times throughout the year associated with anthrax deaths as developed through focus group discussions with NCA pastoralist communities. These seasons correspond roughly with the two dry and two rainy seasons of the year.
 
SNP Profile 
 
A total of 104 single nucleotide polymorphism profiles were identified among the B. anthracis sequences investigated in this study. Profile numbers were assigned arbitrarily. 
 
Genotype_Forde
 
Several of the SNP profiles (n=22) were previously described (Forde et al. 2022), and assigned letters A-V. In the majority of cases, a single SNP profile is linked to a previously-named genotype. However, due to minor differences in the SNPs called between the two studies, there is one instance where a single Genotype (G) now comprises more than one SNP profile.
 
GPS data (Lat/Long)
 
As Microreact does not allow for a mapped representation of data points without complete GPS-data, sequences missing geographical information were arbitrarily assigned the coordinates [-2.378348, 36.039533] for the Microreact project only; this includes the Ames ancestor reference sequence. These coordinates are located in the middle of Lake Natron. No samples were collected there.
For some samples, geographical information was not precise and was limited to the respective region. Therefore, five samples collected in the Arusha region have been assigned the latitude and longitude values of the centre of Arusha city [-3.386925, 36.682995], while two samples from a human patient from near Moshi were assigned the latitude and longitude values of the centre of Moshi [-3.3349, 37.3404] to approximate these sampling locations.
GPS values for human cases have been limited to three decimal positions for reasons of data privacy.
 
 
 
References
 
1.         Ravel J, Jiang L, Stanley ST, Wilson MR, Decker RS, Read TD, et al. The complete genome sequence of Bacillus anthracis Ames "Ancestor". J Bacteriol. 2009;191(1):445-6.
2.         Aminu OR, Forde TL, Ekwem D, Johnson P, Nelli L, Mmbaga BT, et al. Participatory mapping identifies risk areas and environmental predictors of endemic anthrax in rural Africa. Sci Rep. 2022;12(1):10514.
 
