# Supplementary material for: Investigating Bacillus anthracis genomic diversity and trait-specific lineages in an endemic area in northern Tanzania through a combination of traditional and culture-free sequencing approaches
Source: PLoS Negl Trop Dis. 2026 Aug 10;20(8):e0014611. doi: 10.1371/journal.pntd.0014611 (PMC13489486; doi:10.1371/journal.pntd.0014611)
Supplement: Peer review history

# Author comment: Author response to Decision Letter 0

**DOI:** 10.1371/journal.pntd.0014611.r001

**Transfer Alert**

This paper was transferred from another journal. As a result, its full editorial history (including decision letters, peer reviews and author responses) may not be present.

27 Jan 2026

# Peer review: Decision Letter 0

**DOI:** 10.1371/journal.pntd.0014611.r002

**Authors:** Stuart Blacksell, Martin Wainaina

16 Feb 2026

PNTD-D-26-00131Investigating Bacillus anthracis genomic diversity and trait-specific lineages in an endemic area in northern Tanzania through a combination of traditional and culture-free sequencing approachesPLOS Neglected Tropical DiseasesDear Dr. Hilbig,Thank you for submitting your manuscript to PLOS Neglected Tropical Diseases. After careful consideration, we feel that it has merit but does not fully meet PLOS Neglected Tropical Diseases's publication criteria as it currently stands. Therefore, we invite you to submit a revised version of the manuscript that addresses the points raised during the review process.Please submit your revised manuscript by Apr 17 2026 11:59PM. If you will need more time than this to complete your revisions, please reply to this message or contact the journal office at plosntds@plos.org. When you're ready to submit your revision, log on to https://www.editorialmanager.com/pntd/ and select the 'Submissions Needing Revision' folder to locate your manuscript file.Please include the following items when submitting your revised manuscript:* A letter that responds to each point raised by the editor and reviewer(s). You should upload this letter as a separate file labeled 'Response to Reviewers'. This file does not need to include responses to any formatting updates and technical items listed in the 'Journal Requirements' section below.* A marked-up copy of your manuscript that highlights changes made to the original version. You should upload this as a separate file labeled 'Revised Manuscript with Track Changes'.* An unmarked version of your revised paper without tracked changes. You should upload this as a separate file labeled 'Manuscript'.If you would like to make changes to your financial disclosure, competing interests statement, or data availability statement, please make these updates within the submission form at the time of resubmission. Guidelines for resubmitting your figure files are available below the reviewer comments at the end of this letter.We look forward to receiving your revised manuscript.Kind regards,Martin WainainaAcademic EditorPLOS Neglected Tropical DiseasesStuart BlacksellSection EditorPLOS Neglected Tropical Diseases

Shaden Kamhawi

co-Editor-in-Chief

PLOS Neglected Tropical Diseases

orcid.org/0000-0003-4304-636XX

Paul Brindley

co-Editor-in-Chief

PLOS Neglected Tropical Diseases

orcid.org/0000-0003-1765-0002

**Journal Requirements:**

1) Please ensure that the CRediT author contributions listed for every co-author are completed accurately and in full.

At this stage, the following Authors/Authors require contributions: Antonia Hilbig, Matej Medvecky, Tiziana Lembo, Blandina T. Mmbaga, Ireen Kiwelu, Deogratius Mshanga, Shabani K. Motto, Zacharia E. Makondo, Boaz Wadugu, Henri O. Arola, Simo Nikkari, Matthew P. Rubach, John A. Crump, Samantha J. Lycett, Roman Biek, and Taya L. Forde. Please ensure that the full contributions of each author are acknowledged in the "Add/Edit/Remove Authors" section of our submission form.

The list of CRediT author contributions may be found here: https://journals.plos.org/plosntds/s/authorship#loc-author-contributions

2) We noticed that you used the phrase 'data not shown' in the manuscript. We do not allow these references, as the PLOS data access policy requires that all data be either published with the manuscript or made available in a publicly accessible database. Please amend the supplementary material to include the referenced data or remove the references.

3) Some material included in your submission may be copyrighted. According to PLOSu2019s copyright policy, authors who use figures or other material (e.g., graphics, clipart, maps) from another author or copyright holder must demonstrate or obtain permission to publish this material under the Creative Commons Attribution 4.0 International (CC BY 4.0) License used by PLOS journals. Please closely review the details of PLOSu2019s copyright requirements here: PLOS Licenses and Copyright. If you need to request permissions from a copyright holder, you may use PLOS's Copyright Content Permission form.

Please respond directly to this email and provide any known details concerning your material's license terms and permissions required for reuse, even if you have not yet obtained copyright permissions or are unsure of your material's copyright compatibility. Once you have responded and addressed all other outstanding technical requirements, you may resubmit your manuscript within Editorial Manager.

Potential Copyright Issues:

- Figure 4 and 5: Please (a) provide a direct link to the base layer of the map (i.e., the country or region border shape) and ensure this is also included in the figure legend; and (b) provide a link to the terms of use / license information for the base layer image or shapefile. We cannot publish proprietary or copyrighted maps (e.g. Google Maps, Mapquest) and the terms of use for your map base layer must be compatible with our CC BY 4.0 license.

Note: if you created the map in a software program like R or ArcGIS, please locate and indicate the source of the basemap shapefile onto which data has been plotted.

If your map was obtained from a copyrighted source please amend the figure so that the base map used is from an openly available source. Alternatively, please provide explicit written permission from the copyright holder granting you the right to publish the material under our CC BY 4.0 license.

If you are unsure whether you can use a map or not, please do reach out and we will be able to help you. The following websites are good examples of where you can source open access or public domain maps:

* U.S. Geological Survey (USGS) - All maps are in the public domain. (http://www.usgs.gov)

* PlaniGlobe - All maps are published under a Creative Commons license so please cite u201cPlaniGlobe, http://www.planiglobe.com, CC BY 2.0u201d in the image credit after the caption. (http://www.planiglobe.com/?lang=enl)

* Natural Earth - All maps are public domain. (http://www.naturalearthdata.com/about/terms-of-use/).

4) Please amend your detailed Financial Disclosure statement. This is published with the article. It must therefore be completed in full sentences and contain the exact wording you wish to be published.

State what role the funders took in the study. If the funders had no role in your study, please state: "The funders had no role in study design, data collection and analysis, decision to publish, or preparation of the manuscript.".

If you did not receive any funding for this study, please simply state: u201cThe authors received no specific funding for this work.u201d

**Reviewers' comments:** Reviewer's Responses to Questions

**Key Review Criteria Required for Acceptance?**

As you describe the new analyses required for acceptance, please consider the following:

**Methods**

-Are the objectives of the study clearly articulated with a clear testable hypothesis stated?

-Is the study design appropriate to address the stated objectives?

-Is the population clearly described and appropriate for the hypothesis being tested?

-Is the sample size sufficient to ensure adequate power to address the hypothesis being tested?

-Were correct statistical analysis used to support conclusions?

-Are there concerns about ethical or regulatory requirements being met?

Reviewer #1: Yes to all but no ethical or regulatory concerns.

Reviewer #2: The methods are well defined and the appropriate statistical analyses were performed

Reviewer #3: (No Response)

**********

**Results**

-Does the analysis presented match the analysis plan?

-Are the results clearly and completely presented?

-Are the figures (Tables, Images) of sufficient quality for clarity?

Reviewer #1: see below.

Reviewer #2: Results and figures are presented well

Reviewer #3: (No Response)

**********

**Conclusions**

-Are the conclusions supported by the data presented?

-Are the limitations of analysis clearly described?

-Do the authors discuss how these data can be helpful to advance our understanding of the topic under study?

-Is public health relevance addressed?

Reviewer #1: conclusions are fine.

Reviewer #2: Conclusions, along with key caveats are clearly stated. This study is a useful contribution for anthrax surveillance in LMICs

Reviewer #3: (No Response)

**********

**Editorial and Data Presentation Modifications?**

Use this section for editorial suggestions as well as relatively minor modifications of existing data that would enhance clarity. If the only modifications needed are minor and/or editorial, you may wish to recommend “Minor Revision” or “Accept”.

Reviewer #1: see below.

Reviewer #2: (No Response)

Reviewer #3: (No Response)

**********

**Summary and General Comments**

Use this section to provide overall comments, discuss strengths/weaknesses of the study, novelty, significance, general execution and scholarship. You may also include additional comments for the author, including concerns about dual publication, research ethics, or publication ethics. If requesting major revision, please articulate the new experiments that are needed.

Reviewer #1: This study by Hilbig et al. is concerned with the investigation of Bacillus anthracis diversity within the Ngorongoro Conservatory Area and some surrounding areas. Herein, the authors employed classical culturing and array-based DNA enrichment from soil samples of a prominent (hyper)endemic area. Major findings include the successful cultivation as well as enrichment and sequencing of B. anthracis DNA from numerous samples. The authors retrieved an unexpected diversity of SNP genotypes of the pathogen, and bioinformatically characterized this diversity, Notably, a considerable number of samples yielded more than a single SNP genotype.

General comments:

1) The manuscript is very extensive. The authors conducted a detailed analysis of their data. The text does mostly a good job in explaining what has been done and found. However, in my opinion the authors have failed in presenting a capturing story that readers with lower capabilities of deep (bioinformatic) analysis can (or are willing to) follow. This is mostly owed to the authors’ decision to use the (non-canonical) B. anthracis typing nomenclature of Bruce et al. (2020) (reference 23). While this is (of course) not wrong or illegitimate, it is not helpful for easy understanding of the study’s SNP-diversity-topic. The most widely accepted, and in my opinion most useful, canonical SNP-typing system for the analysis at hand, is that of Sahl et al. 2016 (reference 13). Only using this system, coupled with ideal visual (bioinformatic) aids such as minimum spanning trees (MST) based on SNP analysis will provide a straightforward understanding of the central points of the authors’ work. The authors are referred to some examples on how to compile a clear graphical output here: https://pmc.ncbi.nlm.nih.gov/articles/PMC8218748/ (Figs. 2-4), https://pmc.ncbi.nlm.nih.gov/articles/PMC10610006/ (Figs. 2-4), or https://pmc.ncbi.nlm.nih.gov/articles/PMC12566857/ (Figs. 3-6). In such MST figures the SNP-diversity becomes immediately obvious.

2) More, there is no phylogenetic tree showing the placement of the isolates within the global diversity of B. anthracis. This tree can be centered (largely reduced) to the A.Br.034 clade into which the new isolates fall. Also, I suggest collapsing “mini”-lineages of isolates showing less than 5 SNP differences among their closest relatives. This tree can be easily combined into a single figure with the one described in the previous comment

3) This leads me to the second major issue of this manuscript. It is well understood in the B. anthracis research field that differences of only a few (<4, max. 5 SNPs) between isolates are regarded as of low significance (also compare https://pmc.ncbi.nlm.nih.gov/articles/PMC8218748/). Therefore, I would be cautious to call widespread differences of 1-2 SNPs among strains “high genetic diversity”.

4) Though I congratulate the authors for retrieving SNP-typing compatible data from culture-free target-capture, I have considerable reservations for the wide-spread use of this approach for B. anthracis. This is because (and the authors even correctly address this challenge in their Discussion section) there are usually large numbers of closely related Bacillus cereus sensu lato organisms in soil alongside B. anthracis. Often these bacteria feature genomes that are extremely similar to that of B. anthracis. Only long-read genome sequencing techniques can (all but) avoid trans-species DNA assemblies, possibly leading to fictitious SNPs. Read lengths generated in the study at hand were 2x75 or 2x100 bp only, which complicates correct attribution of reads. Indeed, 75 auf of 288 sequences were excluded from further analysis because of quality issues.

5) Finally, because of these challenges it would have been prudent to perform a proof-of-principle experiment beforehand in order to check if different genotypes can be identified by a TC sequencing appraoch in soil at all and at which quality-level. For this, B. anthracis negative soil taken from Ngorongoro would be spiked with spores of two different B. anthracis strains (e.g., Sterne and another vaccine strain such as Pasteur) and genotyped using TC followed by DNA-sequencing.

Specific comments:

6) Line 102: reference 22 should be 13 (A.Br.AncientA was unknown in 2007).

7) L 198: https://pmc.ncbi.nlm.nih.gov/articles/PMC8218748/ could be added as a reference.

8) L 311, better: “…profile of any strain differs…”.

9) L 363-369: I find it problematic to separate anthrax outbreaks by the parameter “time”: spores remain viable in soil for years possible decades and, are thus occasionally the source of recurring outbreaks of the same genotype (give or take a handful of SNPs).

10) 437-438: I find it remarkable that “Forty-four cases were represented by more than one sequence (range: 2-6, median 3)”. But then these sequences only differ in very few SNPs. Again, why should we bother and unnecessarily bloat the SNP-diversity?

11) L 500-503: this mini-paragraph is hard to understand. Please revisit.

12) L511-512: did the authors consider (slightly) higher SNP differences identified in their culture-derived sequences because these organisms were propagated in in-vitro cultures for several extra generations (DNA replications) while the TC-derived sequences were not?

13) Legend of Fig. 1 is odd: “All NCA sequences fell within cluster 3.2 defined by Bruce … corresponding to the 3.1 cluster.” This is Results and thus should not appear in a figure legend.

14) Similar in legend of Fig.2: “While the curve for isolate-derived sequences …sufficiently capture the SNP profile diversity.” This is Results and thus should not appear in a figure legend.

15) Also, header of table 2: “In three of four cases…both methods.” This is Results and thus should not appear in a table header.

16) Finally, in legend of Fig.3: “Fewer SNP differences separate the sequences … over 250 SNPs.” This is Results and thus should not appear in a figure legend.

17) L575 vs last row of Table 4: “28 vs “32” is this correct? Should it not be the same numbers? Did I miss something?

18) L 802 and 816: https://pubmed.ncbi.nlm.nih.gov/23937393/ should be added as a new reference.

Reviewer #2: In this study, using a collection of ~200 anthrax samples collected from human and animal sources in Ngorongoro Conservation Area, the authors examined the genomic diversity as well as the effects of seasonal variation on the spread of BA in the region. The authors also evaluated a culture-free method of sequencing anthrax, bypassing the need for strict biosafety requirements that would otherwise limit BA research in LMICs. The authors found great genomic diversity within the NCA and sometimes even within epidemiologically linked groups. The authors also showed that a culture free target capture method can be useful to estimate BA diversity and build phylogenies. The study is well presented. Major and minor comments below.

## Major

I am curious as to the justification for excluding PXO1 and PXO2 from the target capture. One could make the argument that PXO1 and PXO2 are also 'core' genomic regions in BA though not chromosomal. While it would be a small addition to the total genome sequence length nevertheless genomic diversity between lineages in the PXO1/2 regions would be interesting to investigate. Alternatively, baits capturing non-anthracis bacillus species could also lead to false-positive novel SNP profiles, which can be mitigated by confirming the presence of PXO1/2 regions (Keeping in mind that B. Tropicus for eg may also carry the anthrax plasmid https://www.biorxiv.org/content/10.1101/2023.12.20.572685v1 ).

In addition, some discussion on where the SNP differences between the SNP profiles located on the genome would be useful. Are they randomly distributed or are there hotspots? What is the nature of the mutations (eg: are they mostly non-synonymous?)

L486 - 491: If this rarefaction analysis was performed across all publicly available anthrax genomes, what would be the rate of discovery of new SNP profiles per new genome added? How does that compare to the rate observed in this study for isolate derived and TC-derived? This would contextualize the genomic diversity of BA in the NCA from a global perspective

## Minor

L87: "blood stream" --> "bloodstream"

L259: 111 TC (L233) + 51 (L223) isolates in this study + 72 + 52 from another study totals to 286, not 288. Please double check the numbers and restate them in this section to make it clearer.

L286 - L287: 'Error! Reference source not found' in text

L292 - 293: Outline how only polymorphic sites were extracted from the alignment. Specify the values supplied to the IQ-TREE -fconst parameter

L328 - 336: If I have understood correctly, the authors here created random subsets of TC data and compared the mean SNP distance within each subset against them mean SNP distance between isolate derived sequences. This section can be improved for clarity (maybe a simple flow diagram can be added) as it is currently hard to follow. Was the deduplicated set of sequences used to make the different subsets?

L339: 'Multiple genomes' - Please state exactly how many

Fig 3: Might be better to show all vs. all within-case and between case SNP distances as boxplots

Table 5: Also include between ELG SNP distances

Fig 6: This appears to be the same tree as Fig 1, perhaps the two figures can be combined as shown in Fig S2

L738 - 740: 'In some cases' - please state exactly how many cases. From my understanding four matched samples were obtained out of which 3 showed agreement.

L805: "Inherit limit" - typo, maybe the authors meant "inherent limit" ?

Fig S4: The plot is not labelled correctly so there is no way to tell which is TC and which is isolate derived

Reviewer #3: The article by Antonia Hilbig et al. on the WGS analysis of Bacillus anthracis in an endemic area in northern Tanzania is interesting, original, and suitable for publication in this journal. The manuscript is well written, well organized, and covers important aspects of the current knowledge about anthrax in an endemic country. It complements already published research and is important for enhancing understanding of this significant zoonosis in Africa.

However, some comments need to be addressed before publication.

� In the Introduction, please replace the abbreviation “BA” with B. anthracis.

� The Introduction is too long and should be reduced. For example, in lines 147–174, the authors provide extensive information on risk factors and ecological and environmental aspects, which is not appropriate for the Introduction section. This part should be shortened, and the detailed information should be moved to the Discussion.

• The authors elaborate excessively on the study objectives and attempt to justify each objective in detail. The objectives should be stated more concisely to avoid confusing readers.

• Materials section, line 216: “Additional DNA extracts (n = 5) from animal (4) and human (1) blood samples.” It is unclear whether (4) and (1) refer to reference numbers or to the number of samples. Do you mean (n = 4) and (n = 1), or simply four and one samples? Please clarify and revise accordingly. The same issue appears in Table 1. Please find a way to clearly differentiate reference numbers from sample numbers.

• Figure 2 requires higher resolution.

• The authors report 104 SNP profiles that differed by at least one SNP. Is a difference of one SNP sufficient as a cutoff to distinguish strains? In other bacterial studies, thresholds of 3–5 SNPs are often used. Please justify the selected cutoff or provide supporting references.

• A legend explaining the barplot colors is missing in Figure 4.

• Discussion, lines 718–725: “Our results show that (1) regional clustering…” It is unclear whether the numbering refers to references or to listed points, as both follow the same journal style. Please revise the format (e.g., 1), 1., [1], or a, b, c) to avoid confusion.

• Although the abbreviation TC is used throughout the manuscript, the explanation “targeted-capture (TC)-derived sequences” is provided only in the conclusion. Please define TC at its first occurrence in the manuscript.

**********

PLOS authors have the option to publish the peer review history of their article (what does this mean?). If published, this will include your full peer review and any attached files.

If you choose “no”, your identity will remain anonymous but your review may still be made public.

**Do you want your identity to be public for this peer review?**  For information about this choice, including consent withdrawal, please see our Privacy Policy.

Reviewer #1: No

Reviewer #2: **Yes:**  Vishnu Raghuram

Reviewer #3: No

[NOTE: If reviewer comments were submitted as an attachment file, they will be attached to this email and accessible via the submission site. Please log into your account, locate the manuscript record, and check for the action link "View Attachments". If this link does not appear, there are no attachment files.]**Figure resubmission:**  While revising your submission, we strongly recommend that you use PLOS’s NAAS tool (https://ngplosjournals.pagemajik.ai/artanalysis) to test your figure files. NAAS can convert your figure files to the TIFF file type and meet basic requirements (such as print size, resolution), or provide you with a report on issues that do not meet our requirements and that NAAS cannot fix.

After uploading your figures to PLOS’s NAAS tool - https://ngplosjournals.pagemajik.ai/artanalysis, NAAS will process the files provided and display the results in the "Uploaded Files" section of the page as the processing is complete. If the uploaded figures meet our requirements (or NAAS is able to fix the files to meet our requirements), the figure will be marked as "fixed" above. If NAAS is unable to fix the files, a red "failed" label will appear above. When NAAS has confirmed that the figure files meet our requirements, please download the file via the download option, and include these NAAS processed figure files when submitting your revised manuscript.**Reproducibility:** To enhance the reproducibility of your results, we recommend that authors of applicable studies deposit laboratory protocols in protocols.io, where a protocol can be assigned its own identifier (DOI) such that it can be cited independently in the future. Additionally, PLOS ONE offers an option to publish peer-reviewed clinical study protocols. Read more information on sharing protocols at https://plos.org/protocols?utm_medium=editorial-email&utm_source=authorletters&utm_campaign=protocols

# Author comment: Author response to Decision Letter 1

**DOI:** 10.1371/journal.pntd.0014611.r003

13 Apr 2026

Attachment Submitted filename: Response_to_reviewers.docx

# Peer review: Decision Letter 1

**DOI:** 10.1371/journal.pntd.0014611.r004

**Authors:** Stuart Blacksell, Martin Wainaina

14 May 2026

PNTD-D-26-00131R1Investigating Bacillus anthracis genomic diversity and trait-specific lineages in an endemic area in northern Tanzania through a combination of traditional and culture-free sequencing approachesPLOS Neglected Tropical DiseasesDear Dr. Hilbig,Thank you for submitting your manuscript to PLOS Neglected Tropical Diseases. After careful consideration, we feel that it has merit but does not fully meet PLOS Neglected Tropical Diseases's publication criteria as it currently stands. Therefore, we invite you to submit a revised version of the manuscript that addresses the points raised during the review process.Please submit your revised manuscript by Jun 13 2026 11:59PM. If you will need more time than this to complete your revisions, please reply to this message or contact the journal office at plosntds@plos.org. When you're ready to submit your revision, log on to https://www.editorialmanager.com/pntd/ and select the 'Submissions Needing Revision' folder to locate your manuscript file.Please include the following items when submitting your revised manuscript:* A letter that responds to each point raised by the editor and reviewer(s). You should upload this letter as a separate file labeled 'Response to Reviewers'. This file does not need to include responses to any formatting updates and technical items listed in the 'Journal Requirements' section below.* A marked-up copy of your manuscript that highlights changes made to the original version. You should upload this as a separate file labeled 'Revised Manuscript with Track Changes'.* An unmarked version of your revised paper without tracked changes. You should upload this as a separate file labeled 'Manuscript'.If you would like to make changes to your financial disclosure, competing interests statement, or data availability statement, please make these updates within the submission form at the time of resubmission. Guidelines for resubmitting your figure files are available below the reviewer comments at the end of this letter.As the corresponding author, your ORCID iD is verified in the submission system and will appear in the published article. PLOS supports the use of ORCID, and we encourage all coauthors to register for an ORCID iD and use it as well. Please encourage your coauthors to verify their ORCID iD within the submission system before final acceptance, as unverified ORCID iDs will not appear in the published article. Only the individual author can complete the verification step; PLOS staff cannot verify ORCID iDs on behalf of authors.We look forward to receiving your revised manuscript.Kind regards,Martin WainainaAcademic EditorPLOS Neglected Tropical DiseasesStuart BlacksellSection EditorPLOS Neglected Tropical Diseases

Shaden Kamhawi

co-Editor-in-Chief

PLOS Neglected Tropical Diseases

orcid.org/0000-0003-4304-636XX

Paul Brindley

co-Editor-in-Chief

PLOS Neglected Tropical Diseases

orcid.org/0000-0003-1765-0002

**Journal Requirements:**  We noticed that you used the phrase 'data not shown' in the manuscript. We do not allow these references, as the PLOS data access policy requires that all data be either published with the manuscript or made available in a publicly accessible database. Please amend the supplementary material to include the referenced data or remove the references.**Reviewers' comments:** Reviewer's Responses to Questions

**Key Review Criteria Required for Acceptance?**

As you describe the new analyses required for acceptance, please consider the following:

**Methods**

-Are the objectives of the study clearly articulated with a clear testable hypothesis stated?

-Is the study design appropriate to address the stated objectives?

-Is the population clearly described and appropriate for the hypothesis being tested?

-Is the sample size sufficient to ensure adequate power to address the hypothesis being tested?

-Were correct statistical analysis used to support conclusions?

-Are there concerns about ethical or regulatory requirements being met?

Reviewer #1: No further remarks.

Reviewer #2: (No Response)

**********

**Results**

-Does the analysis presented match the analysis plan?

-Are the results clearly and completely presented?

-Are the figures (Tables, Images) of sufficient quality for clarity?

Reviewer #1: see "Editorial and Data Presentation Modifications".

Reviewer #2: (No Response)

**********

**Conclusions**

-Are the conclusions supported by the data presented?

-Are the limitations of analysis clearly described?

-Do the authors discuss how these data can be helpful to advance our understanding of the topic under study?

-Is public health relevance addressed?

Reviewer #1: No further remarks.

Reviewer #2: (No Response)

**********

**Editorial and Data Presentation Modifications?**

Use this section for editorial suggestions as well as relatively minor modifications of existing data that would enhance clarity. If the only modifications needed are minor and/or editorial, you may wish to recommend “Minor Revision” or “Accept”.

Reviewer #1: The authors have addressed my previous remarks and concerns in their rebuttal. I still disagree with the authors’ reasoning about the negligible relevance of small SNP differences among closely related strains. However, it is fine to have different opinions, as data typically allows for more than a singular explanation. Yet, in my opinion, the most informative visual data interpretation of the work at hand (i.e., supplementary figure 3) lacks any strain designation. Please revisit and add names and SNP distances.

Reviewer #2: (No Response)

**********

**Summary and General Comments**

Use this section to provide overall comments, discuss strengths/weaknesses of the study, novelty, significance, general execution and scholarship. You may also include additional comments for the author, including concerns about dual publication, research ethics, or publication ethics. If requesting major revision, please articulate the new experiments that are needed.

Reviewer #1: see "Editorial and Data Presentation Modifications".

Reviewer #2: The authors have addressed my comments. However for comments #19, #20 (on not including PXO regions in the baits) and #25 (the justification for using +ASC instead of -fconst), while I agree with the authors justifications, I would suggest they also include it in the manuscript itself as a brief sentence (#25 in the appropriate methods section and #19 and #20 in the discussion perhaps?) as I believe they are questions other readers might also have.

**********

PLOS authors have the option to publish the peer review history of their article (what does this mean?). If published, this will include your full peer review and any attached files.

If you choose “no”, your identity will remain anonymous but your review may still be made public.

**Do you want your identity to be public for this peer review?**  For information about this choice, including consent withdrawal, please see our Privacy Policy.

Reviewer #1: No

Reviewer #2: **Yes:**  Vishnu Raghuram

[NOTE: If reviewer comments were submitted as an attachment file, they will be attached to this email and accessible via the submission site. Please log into your account, locate the manuscript record, and check for the action link "View Attachments". If this link does not appear, there are no attachment files.]**Figure resubmission:** While revising your submission, we strongly recommend that you use PLOS’s NAAS tool (https://ngplosjournals.pagemajik.ai/artanalysis) to test your figure files. NAAS can convert your figure files to the TIFF file type and meet basic requirements (such as print size, resolution), or provide you with a report on issues that do not meet our requirements and that NAAS cannot fix.

After uploading your figures to PLOS’s NAAS tool - https://ngplosjournals.pagemajik.ai/artanalysis, NAAS will process the files provided and display the results in the "Uploaded Files" section of the page as the processing is complete. If the uploaded figures meet our requirements (or NAAS is able to fix the files to meet our requirements), the figure will be marked as "fixed" above. If NAAS is unable to fix the files, a red "failed" label will appear above. When NAAS has confirmed that the figure files meet our requirements, please download the file via the download option, and include these NAAS processed figure files when submitting your revised manuscript.**Reproducibility:** To enhance the reproducibility of your results, we recommend that authors of applicable studies deposit laboratory protocols in protocols.io, where a protocol can be assigned its own identifier (DOI) such that it can be cited independently in the future. Additionally, PLOS ONE offers an option to publish peer-reviewed clinical study protocols. Read more information on sharing protocols at https://plos.org/protocols?utm_medium=editorial-email&utm_source=authorletters&utm_campaign=protocols

# Author comment: Author response to Decision Letter 2

**DOI:** 10.1371/journal.pntd.0014611.r005

18 Jun 2026

Attachment Submitted filename: Reviewer_Response_BATNZ_2.docx

# Peer review: Decision Letter 2

**DOI:** 10.1371/journal.pntd.0014611.r006

**Authors:** Stuart Blacksell, Martin Wainaina

26 Jun 2026

PNTD-D-26-00131R2Investigating Bacillus anthracis genomic diversity and trait-specific lineages in an endemic area in northern Tanzania through a combination of traditional and culture-free sequencing approachesPLOS Neglected Tropical Diseases Dear Dr. Hilbig, Thank you for submitting your manuscript to PLOS Neglected Tropical Diseases. After careful consideration, we feel that it has merit but does not fully meet PLOS Neglected Tropical Diseases's publication criteria as it currently stands. Therefore, we invite you to submit a revised version of the manuscript that addresses the points raised during the review process.

Please submit your revised manuscript by Jul 26 2026 11:59PM. If you will need more time than this to complete your revisions, please reply to this message or contact the journal office at plosntds@plos.org. When you're ready to submit your revision, log on to https://www.editorialmanager.com/pntd/ and select the 'Submissions Needing Revision' folder to locate your manuscript file.

Please include the following items when submitting your revised manuscript:* A letter that responds to each point raised by the editor and reviewer(s). You should upload this letter as a separate file labeled 'Response to Reviewers'. This file does not need to include responses to any formatting updates and technical items listed in the 'Journal Requirements' section below.* A marked-up copy of your manuscript that highlights changes made to the original version. You should upload this as a separate file labeled 'Revised Manuscript with Track Changes'.* An unmarked version of your revised paper without tracked changes. You should upload this as a separate file labeled 'Manuscript'.If you would like to make changes to your financial disclosure, competing interests statement, or data availability statement, please make these updates within the submission form at the time of resubmission. Guidelines for resubmitting your figure files are available below the reviewer comments at the end of this letter.

As the corresponding author, your ORCID iD is verified in the submission system and will appear in the published article. PLOS supports the use of ORCID, and we encourage all coauthors to register for an ORCID iD and use it as well. Please encourage your coauthors to verify their ORCID iD within the submission system before final acceptance, as unverified ORCID iDs will not appear in the published article. Only the individual author can complete the verification step; PLOS staff cannot verify ORCID iDs on behalf of authors.

We look forward to receiving your revised manuscript.Kind regards,

Martin Wainaina

Academic EditorPLOS Neglected Tropical DiseasesStuart BlacksellSection Editor PLOS Neglected Tropical Diseases Shaden Kamhawi

co-Editor-in-Chief

PLOS Neglected Tropical Diseases

orcid.org/0000-0003-4304-636XX

Paul Brindley

co-Editor-in-Chief

PLOS Neglected Tropical Diseases

orcid.org/0000-0003-1765-0002

**Additional Editor Comments (if provided):**

I thank the authors for their substantial work on this important topic.

I would like a clarification before taking this forward: please confirm whether the GPS coordinates for human cases in the study were masked to avoid potentially identifying information.

If so, I request the authors to state this, perhaps in the supplementary readme file under the GPS data paragraph.

If not, please use appropriate masking of the ones in the Ngorongoro wards, e.g. by making them a bit coarser/report at ward level, etc. **Reviewers' comments:** Reviewer's Responses to Questions

**Key Review Criteria Required for Acceptance?**

As you describe the new analyses required for acceptance, please consider the following:

**Methods**

-Are the objectives of the study clearly articulated with a clear testable hypothesis stated?

-Is the study design appropriate to address the stated objectives?

-Is the population clearly described and appropriate for the hypothesis being tested?

-Is the sample size sufficient to ensure adequate power to address the hypothesis being tested?

-Were correct statistical analysis used to support conclusions?

-Are there concerns about ethical or regulatory requirements being met?

Reviewer #1: (No Response)

**********

**Results**

-Does the analysis presented match the analysis plan?

-Are the results clearly and completely presented?

-Are the figures (Tables, Images) of sufficient quality for clarity?

Reviewer #1: (No Response)

**********

**Conclusions**

-Are the conclusions supported by the data presented?

-Are the limitations of analysis clearly described?

-Do the authors discuss how these data can be helpful to advance our understanding of the topic under study?

-Is public health relevance addressed?

Reviewer #1: (No Response)

**********

**Editorial and Data Presentation Modifications?**

Use this section for editorial suggestions as well as relatively minor modifications of existing data that would enhance clarity. If the only modifications needed are minor and/or editorial, you may wish to recommend “Minor Revision” or “Accept”.

Reviewer #1: (No Response)

**********

**Summary and General Comments**

Use this section to provide overall comments, discuss strengths/weaknesses of the study, novelty, significance, general execution and scholarship. You may also include additional comments for the author, including concerns about dual publication, research ethics, or publication ethics. If requesting major revision, please articulate the new experiments that are needed.

Reviewer #1: (No Response)

**********

PLOS authors have the option to publish the peer review history of their article (what does this mean?). If published, this will include your full peer review and any attached files.

If you choose “no”, your identity will remain anonymous but your review may still be made public.

**Do you want your identity to be public for this peer review?**  For information about this choice, including consent withdrawal, please see our Privacy Policy.

Reviewer #1: No

[NOTE: If reviewer comments were submitted as an attachment file, they will be attached to this email and accessible via the submission site. Please log into your account, locate the manuscript record, and check for the action link "View Attachments". If this link does not appear, there are no attachment files.] **Figure resubmission:**   While revising your submission, we strongly recommend that you use PLOS’s NAAS tool (https://ngplosjournals.pagemajik.ai/artanalysis) to test your figure files. NAAS can convert your figure files to the TIFF file type and meet basic requirements (such as print size, resolution), or provide you with a report on issues that do not meet our requirements and that NAAS cannot fix.

After uploading your figures to PLOS’s NAAS tool - https://ngplosjournals.pagemajik.ai/artanalysis, NAAS will process the files provided and display the results in the "Uploaded Files" section of the page as the processing is complete. If the uploaded figures meet our requirements (or NAAS is able to fix the files to meet our requirements), the figure will be marked as "fixed" above. If NAAS is unable to fix the files, a red "failed" label will appear above. When NAAS has confirmed that the figure files meet our requirements, please download the file via the download option, and include these NAAS processed figure files when submitting your revised manuscript. **Reproducibility:**  To enhance the reproducibility of your results, we recommend that authors of applicable studies deposit laboratory protocols in protocols.io, where a protocol can be assigned its own identifier (DOI) such that it can be cited independently in the future. Additionally, PLOS ONE offers an option to publish peer-reviewed clinical study protocols. Read more information on sharing protocols at https://plos.org/protocols?utm_medium=editorial-email&utm_source=authorletters&utm_campaign=protocols

# Author comment: Author response to Decision Letter 3

**DOI:** 10.1371/journal.pntd.0014611.r007

17 Jul 2026

Attachment Submitted filename: Reviewer_Response_BATNZ_4.docx

# Editor report: Decision Letter 3

**DOI:** 10.1371/journal.pntd.0014611.r008

**Authors:** Stuart Blacksell, Martin Wainaina

22 Jul 2026

Dear PhD Hilbig,

We are pleased to inform you that your manuscript 'Investigating Bacillus anthracis genomic diversity and trait-specific lineages in an endemic area in northern Tanzania through a combination of traditional and culture-free sequencing approaches' has been provisionally accepted for publication in PLOS Neglected Tropical Diseases.

Before your manuscript can be formally accepted you will need to complete some formatting changes, which you will receive in a follow up email. A member of our team will be in touch with a set of requests.

Please note that your manuscript will not be scheduled for publication until you have made the required changes, so a swift response is appreciated.

IMPORTANT: The editorial review process is now complete. PLOS will only permit corrections to spelling, formatting or significant scientific errors from this point onwards. Requests for major changes, or any which affect the scientific understanding of your work, will cause delays to the publication date of your manuscript.

Should you, your institution's press office or the journal office choose to press release your paper, you will automatically be opted out of early publication. We ask that you notify us now if you or your institution is planning to press release the article. All press must be co-ordinated with PLOS.

Thank you again for supporting Open Access publishing; we are looking forward to publishing your work in PLOS Neglected Tropical Diseases.

Best regards,

Martin Wainaina

Academic Editor

PLOS Neglected Tropical Diseases

Stuart Blacksell

Section Editor

PLOS Neglected Tropical Diseases

Shaden Kamhawi

co-Editor-in-Chief

PLOS Neglected Tropical Diseases

orcid.org/0000-0003-4304-636XX

Paul Brindley

co-Editor-in-Chief

PLOS Neglected Tropical Diseases

orcid.org/0000-0003-1765-0002

***********************************************************

# Editor report: Acceptance letter

**DOI:** 10.1371/journal.pntd.0014611.r009

**Authors:** Stuart Blacksell, Martin Wainaina

Dear PhD Hilbig,

We are delighted to inform you that your manuscript, "Investigating Bacillus anthracis genomic diversity and trait-specific lineages in an endemic area in northern Tanzania through a combination of traditional and culture-free sequencing approaches," has been formally accepted for publication in PLOS Neglected Tropical Diseases.

We have now passed your article onto the PLOS Production Department who will complete the rest of the publication process. All authors will receive a confirmation email upon publication.

The corresponding author will soon be receiving a typeset proof for review, to ensure errors have not been introduced during production. Please review the PDF proof of your manuscript carefully, as this is the last chance to correct any scientific or type-setting errors. Please note that major changes, or those which affect the scientific understanding of the work, will likely cause delays to the publication date of your manuscript. Note: Proofs for Front Matter articles (Editorial, Viewpoint, Symposium, Review, etc...) are generated on a different schedule and may not be made available as quickly.

Soon after your final files are uploaded, the early version of your manuscript will be published online unless you opted out of this process. The date of the early version will be your article's publication date. The final article will be published to the same URL, and all versions of the paper will be accessible to readers.

For Research Articles, you will receive an invoice from PLOS for your publication fee after your manuscript has reached the completed accept phase. If you receive an email requesting payment before acceptance or for any other service, this may be a phishing scheme. Learn how to identify phishing emails and protect your accounts at https://explore.plos.org/phishing.

Thank you again for supporting open-access publishing; we are looking forward to publishing your work in PLOS Neglected Tropical Diseases.

Best regards,

Shaden Kamhawi

co-Editor-in-Chief

PLOS Neglected Tropical Diseases

Paul Brindley

co-Editor-in-Chief

PLOS Neglected Tropical Diseases
